# Strengthening the strategy to sustain optimal iodine status in the Republic of Moldova: Assessing the use of iodized salt in industrially processed foods

**DOI:** 10.1371/journal.pone.0289142

**Published:** 2023-07-27

**Authors:** Ion Salaru, Daniela Demiscan, Lilia Turcan

**Affiliations:** 1 National Public Health Agency of the Republic of Moldova, Chişinău, Moldova; 2 Ministry of Health, Labour and Social Protection of the Republic of Moldova, Chişinău, Moldova; 3 Iodine Global Network, Seattle, Washington, United States of America; Public Library of Science, UNITED KINGDOM

## Abstract

**Background:**

Although national legislation in the Republic of Moldova includes the use of iodized salt in processed food industry as well as household (cooking and table) salt, little is known of the actual use of iodized salt in the food industry and its contribution to the overall iodine intake of the population. This study has helped to address the gap in understanding about how much iodized salt is used in the production of different foods and to identify where more information is still required in order to more fully characterize the contribution of food industry salt to population iodine intake.

**Methods:**

Using the available food consumption and production data, the assessment introduces a novel modelling technique, based on the methodology of the IGN Programme Guidance to assess the use of iodized salt in industrially produced foods (IPF). The method included the identification of key salt containing IPF and modelling of the salt and potential iodine intake in two groups of the population, non-pregnant adults and pregnant women. The findings were synthetized to develop a list of recommendations for adjustment or strengthening the existing salt iodization strategy.

**Main results:**

In RM, the salt used for industrially produced bread and household use provide almost 80% of all salt intake for adults. The intake from iodized salt at household level and the 8 key salt- containing IPF is estimated to currently meet 89% and 53% of the recommended nutrient for adults and pregnant women, respectively. If all salt used at household level and industrial bread baking would be iodized, then, potentially, it could ensure 181% and 109%, respectively, of the required iodine intake.

**Conclusions:**

Use of iodized salt in the processed food industry is of growing significance and universal use of iodized salt at household level and in bread production could result in a desirable increase in iodine intake. The national salt iodization strategy should include strengthened regulatory monitoring of iodized salt use in the bread baking industry.

## Background

The Republic of Moldova is situated on the south-eastern border of the European Union and neighbours with Romania on the west and Ukraine on the east and is home to 2.71 million people. With a gross development product (GDP) per capita of US$ 2,006 it is classified as a low-middle income country. The economic growth of the recent years has contributed to improved health outcomes, with life expectancy returning to pre-independence levels of the 1990s, 77 years for women and 68 years for men, although still remains low in comparison to the European Union average of 83.7 and 78 years, respectively [1]. Although the Republic of Moldova performs well against other developing countries, it continues to experience undernutrition among its under-five children, adolescents and pregnant women and faces increasing obesity rates [2].

Evidence of iodine deficiency was documented in The Republic of Moldova in the mid-90s [3] and a national program to prevent iodine deficiency was initiated in 1998 [4]. To ensure continued implementation of salt iodization, three consecutive dedicated national programs were enacted by the Government of Moldova. Starting from 2021, nutrition interventions will come under the umbrella of the national program to prevent non-communicable diseases. In 2011, national regulation was put in place for mandatory use of iodized salt in households, public catering and by the food industry, stipulating a standard concentration of 20 ± 15 mg/kg iodine [4].

A survey in 2016 showed the iodine status of the adult population was adequate at national level with a median urinary iodine concentration (MUIC) of 136 μg/L among adults aged 18 to 65 years, and no large difference in MUIC between urban and rural residence or gender [5]. 77% of households reported using iodized salt, with 57% utilizing adequately iodized salt (iodine content of ≥15mg/kg) [5].

Reported household utilization of iodized salt varied greatly between residence areas and socioeconomic groups of population and was significantly lower in rural areas and less affluent groups, 44.5% and 50.6% compared to 77.9% and 80.9%, respectively. This indicated that rural and poorer households had less access to iodine intake from iodized salt. Iodine status among school age children and pregnant women was studied in 2012 and a MUIC of 204 μg/l and 173 μg/l, respectively, at national level indicated adequate status [3]. Little is known of the iodized salt use and iodine status in the auto-proclaimed independent region on the left bank of the river Nistru where 0.5 million people reside.

Positive program results were achieved in the use of iodized salt for preschool and school feeding services as all kindergartens and primary schools use iodized salt only [6]. The uptake of iodized salt by the food industry is inconsistent and, by 2018, the practice was consistently sustained by few large bakeries only, as food producers remained reluctant to use iodized salt because of perceived negative effects of iodized salt on product quality. Inconsistent regulatory monitoring meant there were few checks on its use by the food industry.

Two national studies, the Dietary Salt Intake Survey in 2016 [7] and the Food Environment Survey for The Republic of Moldova 2016 [8] conducted with support from WHO Euro provided evidence that a significant proportion of salt consumption comes from processed foods. In light of the findings, National Food and Nutrition Program stakeholders felt the need for better understanding of the potential contribution of processed foods to the salt and iodine intake of the population. This type of evidence was felt necessary for advocacy and re-engagement of food industry and to expand awareness of the use of fortified foods, as public’ interest for healthy foods and diets was growing in the country.

In recognition of similar needs globally, The Iodine Global Network (IGN) had developed *“Programme Guidance—Assessing the Use of Iodized Salt in Industrially Processed Foods”* and requested expression of interest from national teams to participate in pilot implementation of the draft Guidance (for more details please see Introductory paper to the special collection. The Ministry of Health, Labour and Social Protection (MHLSP) of the Republic of Moldova and National Public Health Agency (NPHA), with support from the IGN National Coordinator, submitted an expression of interest letter and were selected to participate in the pilot, along with four other countries globally. Implementation activities started in March 2019.

## Methodology

There were three main aspects of this study. Firstly, for the first time, through reviewing dietary information, obtaining information from food industries and modelling, the potential and estimated current contribution of household salt and processed food salt respectively to the iodine intake of population. Secondly, to conduct a brief situational analysis in the light of the findings from the modelling exercise and determine the need to strengthen or expand the existing strategy for salt iodization, to include salt for all key salt-containing IPF where this is found to be needed. Thirdly, to propose improvements to the implementation of salt iodization strategy given that salt iodization is mandated but believed to be poorly enforced at food processing.

### Stakeholders involved

A national working group was established, drawing from the ranks of the technicians collaborating under the National Food and Nutrition Program (NFNP) 2014–2020 (Governmental Decree nr. 730 of 2014) and included representatives of the MHLSP, NPHA, National Food Security Agency, salt importers and Department of Food Technology at State Technical University of Moldova. The group was assisted by a small technical team inclusive of the head of the non-communicable disease prevention department of the NPHA and IGN National Coordinator, with oversight provided by the Head of the Public Health Policy Department of the MHLSP, acting as the Secretary of the National Coordination Council of the NFNP 2014–2020.

### Process

The national working group acted in accordance with the list of responsibilities outlined in the Guidance document. Concise action and budget plans were developed and implementation was monitored against the time targets set out. The piloting phase took place from March until December 2019 and concluded with a consultative workshop in February 2020 and final assessment report, inclusive of a list of recommendations for a strengthened national salt iodization strategy. Throughout the process, technical assistance was provided by IGN Guidance technical support team through means of calls, emails and follow-up, as well as review of outcomes of modelling and the content of the assessment report.

In the first step of the modelling exercise, an assessment of availability of data sources was conducted, using a mixed approach, combining verification of available data from surveys, retail prices, official databases and requests to governmental and industry bodies for additional data, as follows:

1. Identification of key industrially produced foods (IPF) contributing to salt intake, the Food Environment Survey for Moldova 2016 [8] was confirmed to include data on salt content of 20+ foods, as well as information on average portion sizes and frequency of IPF consumption by adults aged 18+ years of age, residing in the capital city/urban area. Salt content of IPF was additionally verified through checks of food nutrition labels in the supermarkets and by referencing the national food standards.
2. Estimates of adult per capita consumption of identified key salt-containing IPF were obtained from annual national budget household surveys published by the National Bureau of Statistics (NBS) [9].
3. Data on production volumes of identified key salt-containing IPF was obtained through direct request to the NBS.
4. Data on imports of foods: (a) volumes of food grade non-iodized and iodized salt were obtained through direct request to the national Customs Department; (b) while data on volumes or type of imported key salt containing IPF would have been useful, given that imported IPF constitute a significant portion of food supply for the country and could contain iodized salt if manufactured with iodized salt, it was not possible to obtain such information at the time of our assessment.

In the next step of the assessment, we selected the appropriate models and scenarios from the programme guidance to estimate the current and potential intake of iodine through IPF. Based on available data for adult per capita consumption of IPF and their salt content, it was possible to model the potential and estimated current likely contribution of selected IPF to iodine intake for two population groups, non-pregnant adults and pregnant women. The model for pregnant women was based on the assumption that food consumption among this group was the same as for non-pregnant adults, since no population specific consumption data for pregnant women were available in the country.

The team investigated three potential scenarios within these models: (1) potential iodine intake when all food grade salt (100%) is iodized; (2) estimated current iodine intake (based on current evidence of the 77% utilization of iodized salt in households, 50% use of iodized salt at bakeries (public health expert estimation), 20% use of iodized salt in pasta production (estimated based on volume of imports from countries known to legislate for the use of iodized salt by the food industry), 12% for pickles and 0% for other foods); and (3) potential iodine intake if current iodization levels are maintained and a national salt reduction target of 30% is achieved. This last scenario was based on the assumption that 30% salt reduction is achieved for household salt consumption and consumption of salt from all selected IPF.

The formula used for estimating the potential iodine intake from household salt and each IPF except bread is as follows: Estimated average percent dry weight salt content of the IPF × mean of the national salt iodine standard (27.5 mg/kg) × estimated average daily per capita consumption of the household salt or the selected IPF x 70% (to account for up to 30% loss of iodine in the final consumed product). The same formula was used to estimate potential iodine intake from bread, except that the value for salt iodine used was 37.5 mg/kg, based on information from the largest national bakery.

## Results

The assessment to identify key salt-containing IPF resulted in the inclusion of 8 foods, along with household salt. The list of these foods is presented in Table 1 along with information on typical intake, the salt content, and potential and estimated current iodine intake (according to scenarios 1 and 2). Household salt, added to meals and used in cooking, was included in the model as national surveys indicate that a large proportion of adults use household salt daily or frequently during the week [7, 8].

**Table 1:** Final list of IPF selected for modelling, with determined salt content, typical consumption estimates, and estimated contribution to salt and iodine intake.

Household salt and salt containing IPF	Estimated average daily per capita consumption (g)^a^	Salt content (% product weight)^b^	Estimated daily salt intake from the product (g)	Potential iodine intake (μg) if 100% food grade salt iodized	Estimated current iodine intake (μg) based on data for current salt iodization^c^
Household salt	3.5	100.0%	3.6	69	53
Bread and baked goods	315	1.9%	5.8	153	77
Sausages (boiled)	17	1.9%	0.3	6	0
Cheese (hard)	7	1.9%	0.1	2	0
Cheese (brynza)*	7	3.5%	0.2	4	0
Dried fish	8	3.3%	0.3	5	0
Instant noodles**	10	4.7%	0.5	9	0
Pasta (dried)	21	2.5%	0.5	10	2
Pickles (in brine)	25	2.5%	0.6	12	1
Total consumption/ salt intake	412	-	11.9	271	123

^a^ Average daily intake was determined using data from the Households budget report from the National Bureau of Standards

^b^ Salt content was based on data from the Food Environment Survey for Moldova 2016: Technical report. 2018. WHO Euro

^c^ Based on the following percent iodized salt in each product: 77% for household salt, 50% of salt in bread, 20% of salt in imported pasta products, 12% of salt in pickles, and 0% of salt in all other products.

* According to Government of RM Regulation on quality of milk and dairy produce (HG nr. 158 din 2019), the threshold of salt content for brynza (in fresh and matured brine) is set rather high: 2 to 6g/100gr produce; and given which we proposed the level of 3.5%, as an average

** Reflects actual salt content, as calculated from actual sodium content obtained in at Food Environment Study (1485mg/portion of 80gr) converted to 4.65gr salt (http://oxsalt.org.uk/db/?page_id=37#sodiumtosaltcalculator)

Data show that the total average daily adult per capita salt intake from household salt and the selected IPF in the Republic of Moldova constitutes 11.9g, of which 5.8g comes from bread and baked goods and 3.6g from household salt with a further 2.5g from selected IPF other than bread (combined contribution). The estimated average daily per capita salt intake is slightly higher than that reported in an earlier national survey which estimated adult salt consumption of 10.8+/-4.9g/day [7].

Furthermore, the modelling showed that 181% and 109% of the Recommended Nutrient Intake (RNI) for iodine could potentially be met for adults and for pregna, respectively, if 100% of household and food industry salt for the 8 key salt-containing IPF was iodized (scenario 1).

Based on current estimates for the use of iodized salt at households and in the selected IPF (see methods), these foods are estimated to contribute to 89% and 53% of the RNI for non-pregnant adults, and pregnant and lactating women, respectively (scenario 2).

If the 30% salt reduction target is met and the use of iodized salt remained at the same level as current estimates, then these same sources of iodized salt would only meet 62% and 37% of the daily iodine RNI for non-pregnant adults, and pregnant and lactating women, respectively (scenario 3).

**Fig 1:**
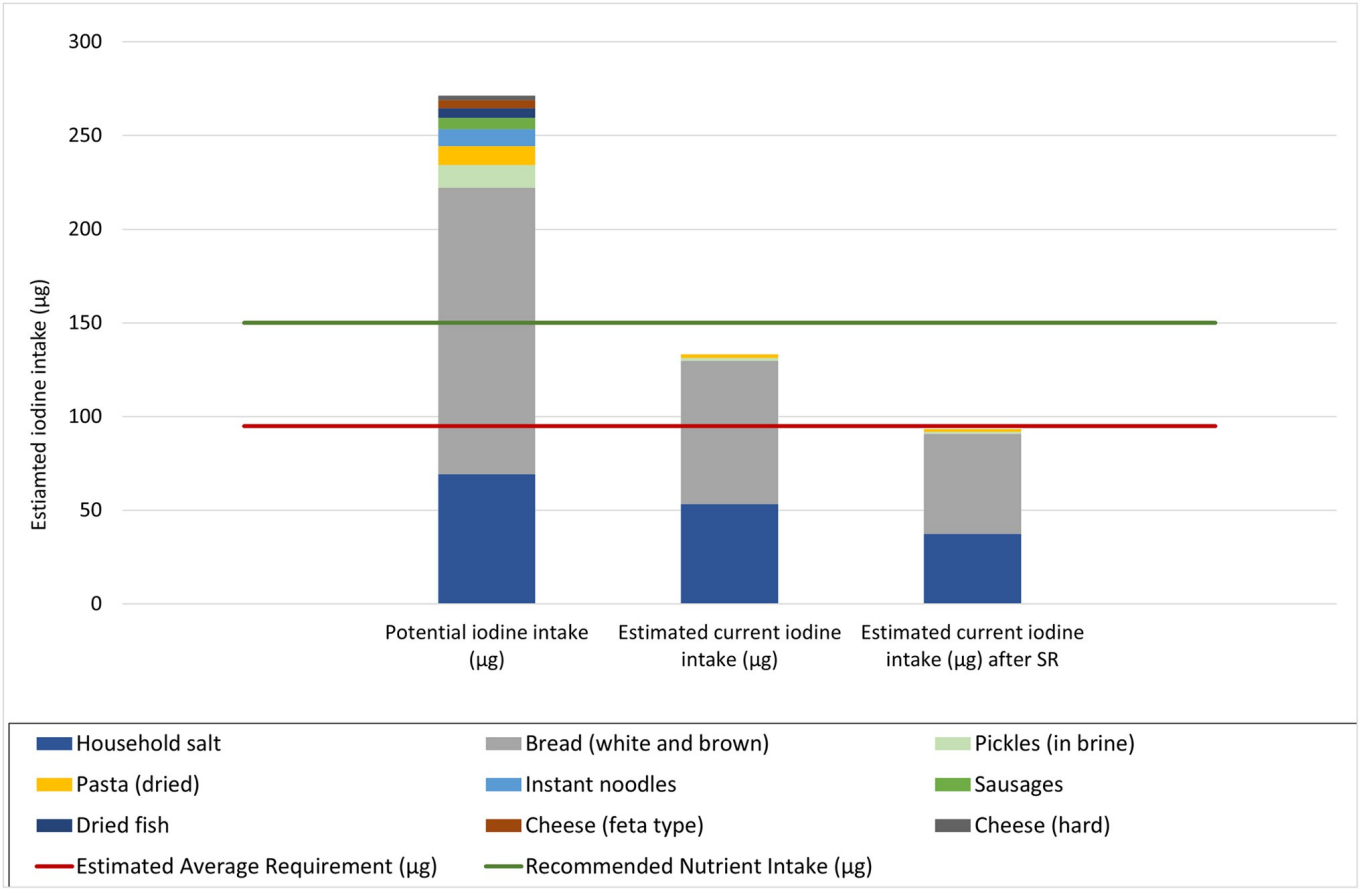
Potential (all food grade salt iodized) and estimated current (based on actual percent salt iodized) contribution of household salt and salt in the selected IPF to percent RNI for iodine for non-pregnant adults, and the likely impact of a 30% reduction in salt intake to estimated current iodine intake from these foods.

Fig 1 illustrates the estimated contribution to the RNI for iodine among non-pregnant adults for the three scenarios modelled.

Our assessment demonstrates that the use of adequately iodized salt by the food industry can make a significant contribution to dietary iodine intake. The findings show that in The Republic of Moldova the current main sources of iodized salt in the diet for non-pregnant adults are household salt and salt in bread and other baked goods, contributing, 36% and 51%, respectively, to the daily adult RNI for iodine under scenario 2 (current estimated levels of salt iodization). The relatively high proportion of iodine intake from iodized salt used in the bakery industry despite the fact that only 50% of bakery salt is currently estimated to be iodized, can be explained by the relatively high dietary intake (shown in Table 1) and the fact that bakery salt could potentially have a higher iodine content that edible table salt. Bread is a staple food in the country, both in urban and rural populations. If all bread and bakery goods were produced using iodized salt, this could meet 102% of the RNI for iodine for adults.

## Discussion

The findings and recommendations of the assessment were discussed at a national workshop held at the NPHA offices on February 28, 2020. Based on feedback received, the team provided a final version of the assessment report to the Ministry of Health. Using the findings outlined in the final national report, the NPHA revised the national Salt Regulation (2011), which now includes provision for mandatory use of iodized salt at baking industry as well as food catering services provided by the state, such as kindergartens, schools, hospitals, social centers, and army.

The choice of bread as the most appropriate food vehicle for salt iodization is justified by the fact that it is eaten regularly by all population groups, including women of childbearing age and young children across all socio-economic sub-groups. Mandatory iodization of salt for IPF, including, bakery salt, is applied in countries such as Belarus, Denmark, the Netherlands, and Switzerland, and has led to a desirable increase in the population’s iodine intake. In Belarus, bread baked with iodized salt meets 40% to 70% of the daily iodine requirement [10].

### Need to strengthen or expand the existing strategy for salt iodization

Given the combination of some critical contextual factors, such as low availability of adequately iodized salt at household level (<60%), low estimated proportion of bread baked with iodized salt and the national target to reduce salt consumption by 30%, there is a clear need to strengthen the implementation of the existing salt iodization strategy. Reinforcement of regulatory monitoring, improved and updated monitoring of iodine status in vulnerable populations such as pregnant women, and harmonization of salt iodization and salt reduction effort for the prevention of non-communicable diseases are important next steps.

### Lessons learned

A unique aspect of this work is that modelling of salt and potential iodine intake based on food consumption data have not previously been used in the Republic of Moldova. The methodology could be used periodically to measure these important monitoring indicators of the salt iodization strategy, especially as new consumption data are available and / or if salt reduction strategies become successful; therefore, the methodology holds potential for improved program planning.

Additionally, it became clear that there is a need for clarification of the responsibility for the regulatory monitoring of fortified foods, including iodized salt. A new Food Security Law (Legea Securitatii Alimentare) was endorsed through the Government Decree nr. 306 in 2018 and entered into force in February 2020 [11], under which the monitoring of fortified foods, (including, iodized salt) was assigned to the newly created National Food Security Agency and the transfer of responsibility from NPHA is not yet complete. The assessment serves as a valuable tool for advocacy, analysis and planning of national food fortification as part of larger nutrition and diet related programming.

## Conclusion

This study highlighted the relevance and importance of iodized salt in processed foods for contributing to iodine intake in the Republic of Moldova, where salt in diets is increasingly sourced from processed foods. The results of our modelling demonstrated that given the significant share of salt from bread in overall salt consumption, and the relatively uniform levels of bread intake among all socio-economic groups in the country, the full and rapid replacement of common salt with iodized salt in the baking industry can ensure a sustainable increase in the iodine intake of the population.

Evidence of iodine intake from significant dietary sources of salt in combination with data from a recent population iodine status survey allows us to determine that dietary iodine intake is currently sufficient for overall adequate iodine status. The data from both studies also confirmed that, at present, there is no need to adjust the salt iodine standard. However, consistent access to adequately iodized salt through regulatory monitoring and enforcement of the use of only iodized salt by households, by the baking industry and in state meal catering services is critical. Once the proposed salt reduction target is achieved, potentially through reformulation of IPF, the appropriateness of the iodine level of food grade salt should be assessed based on continuous monitoring of population iodine status, especially in pregnant and lactating women, adolescent girls and young children.

At the same time, improved availability of national data on IPF and household salt consumption and on the level and type of salt used in these products is needed. This type of data could result from an increased strategic focus on regulatory monitoring and reporting of iodized salt at import, retail, and in the bread industry as part of the new national NCD Program 2021–2025. These data could be complimented by data on household use of iodized salt, as part of the Nutrition and Diet component of this program.

Other measures to be taken include heightened awareness of food producers around the use of iodized salt and food reformulation in line with the national target of 30% reduction of salt content.

### Assessment limitations

While adequate iodine nutrition status has been confirmed in the Republic of Moldova, the estimated current contribution of iodized salt in household salt and selected IPF to adult iodine intake was only 89% of the RNI. A possible explanation for how the remainder of iodine requirements are met could come from an underestimation of the iodized salt use in the baking industry as the actual use could be higher than the 50% of the industry estimated by us. Concomitantly, other IPF could be manufactured using iodized salt but have been not included in this assessment, for example, at least two large ice cream producers in the country use iodized salt. In addition, imported IPF included or not included in this assessment could be a greater source of iodized salt than currently realized. For example, food condiments, such as flavourings, seasonings, spices, bouillon cubes, stock cubes, broth cubes, are known to be salty, and even possibly prepared with iodized salt. These are believed to be widely consumed in Moldova however there are very limited publicly available survey-based data to indicate with certainty the type, amounts or frequency of consumption. The lack of such data creates a limitation to the overall study of the salt/ iodized salt consumption. There are also likely to be variable non-salt sources of iodine from drinking water and other foods. To close the data gap and as a follow up to this assessment, the team recommended to work directly with largest food producers to collect data on their use of iodized salt in food manufacturing.

In addition, the estimates for the potential impact of achieving salt reduction targets on salt and, therefore, iodine intake, were based on a reduction of 30% across all sources of salt. In reality, reductions from different salt sources would be expected to vary based on feasibility of respective behaviour change in consumption and of product reformulation. However, the model still provided a helpful indication of how salt reduction may affect iodine intake.

## Supporting information

S1 Data (PDF)

S1 File (XLSX)
