# Correction: Strengthening the strategy to sustain optimal iodine status in the Republic of Moldova: Assessing the use of iodized salt in industrially processed foods

**DOI:** 10.1371/journal.pone.0296373

**Published:** 2023-12-20

**Authors:** Ion Salaru, Daniela Demiscan, Lilia Turcan

The S1 File (XLSX) was published incorrectly. Please omit this file when reading the article.

## References

[1] Salaru I, Demiscan D, Turcan L. (2023) Strengthening the strategy to sustain optimal iodine status in the Republic of Moldova: Assessing the use of iodized salt in industrially processed foods. PloS ONE, 18(7): e0289142. 10.1371/journal.pone.0289142 37498852 PMC10374049

