# Supplementary material for: Strengthening the strategy to sustain optimal iodine status in the Republic of Moldova: Assessing the use of iodized salt in industrially processed foods
Source: PLoS One. 2023 Jul 27;18(7):e0289142. doi: 10.1371/journal.pone.0289142 (PMC10374049; doi:10.1371/journal.pone.0289142)
Supplement: S1 Data — (PDF) [file pone.0289142.s001.pdf]

Data shown is for non-pregnant adults RNI =150ug iodine
For pregnant women, the same intake data were used, but the RNI for iodine is 250ug

						Main Outcome: If all food grade salt is iodised at mean mg/kg of the national salt iodine standard (adjusted for imports as needed), and assuming 30% loss of iodine				Optional - estimated current iodine intake: If data on current household and food industry use of iodised salt known. Based on estimated current % salt iodised with assumed mean (mg/kg) national salt iodine standard (adjusted for imports as needed) and 30% iodine loss					Optional - iodine intake if salt reduction (SR) achieved with variable SR targets by food type, if known. Based on *potential* iodine intake if all food grade salt iodised (O) and on iodine intake with *estimated current* % salt iodised (P).		
**Column label:**		**A**	**B**	**C**	**D**	**E**	**F**	**G**	**H**	**I**	**J**	**K**	**L**	**M**	**N**	**O**	**P**
**Food product (aim for at least 4 products). Include household (HH) salt where possible**	**Source reference code**	**Estimated average daily per capita consumption (g)**	**Salt content (% product weight)**	**Estimated daily salt intake from the product (g)**	**Salt iodine standard (mg/kg or µg/g)** Overwrite the autofill where different standard applies, e.g. for imported foods	**Potential iodine intake (µg) from daily intake (g) of the product**	**Potential % EAR for iodine from typical daily intake of the product**	**Potential % RNI for iodine from typical daily intake of the product**	**Potential % UL for iodine from typical daily intake of the product**	**Estimated percent of total salt used in the product that is iodised**	**Estimated current iodine intake (µg) from daily intake of the product (g)**	**Estimated current % EAR for iodine from typical daily intake of the product**	**Estimated current % RNI for iodine from typical daily intake of the product**	**Estimated current % UL for iodine from typical daily intake of the product**	**Target reduction in salt content of the product (%)**	**Potential iodine intake (µg) from daily intake of the product (g) based on ALL salt iodised and after SR**	**Estimated iodine intake (µg) from daily intake of the product (g) based on estimated current levels iodisation and after SR**
**Calculation for each column:**				A * B		C * D * 70% (for losses)	E / EAR iodine for population group	E / RNI iodine for population group	E / UL iodine for population group		E * I	J / EAR iodine for population group	J / RNI iodine for population group	J / UL iodine for population group		E * N	J * N
**Food type**						**Potential iodine intake (µg)**	**Potential % EAR iodine**	**Potential % RNI iodine**	**Potential % UL iodine**		**Estimated current iodine intake (µg)**	**Current % EAR iodine**	**Current % RNI iodine**	**Current % UL iodine**		**Potential iodine intake (µg) after SR**	**Estimated current iodine intake (µg) after SR**
Household salt	1	3.6	100.0%	3.6	27.5	69.3	72.9%	46.2%	11.6%	77%	53.4	56.2%	35.6%	8.9%	30%	48.5	37.4
Bread (white and brown)	2	315.0	1.9%	5.8	37.5	153.0	161.0%	102.0%	25.5%	50%	76.5	80.5%	51.0%	12.7%	30%	107.1	53.5
Pickles (in brine)	1	25.0	2.5%	0.6	27.5	12.0	12.7%	8.0%	2.0%	12%	1.4	1.5%	1.0%	0.2%	30%	8.4	1.0
Pasta (dried)	1	21.0	2.5%	0.5	27.5	10.1	10.6%	6.7%	1.7%	20%	2.0	2.1%	1.3%	0.3%	30%	7.1	1.4
Instant noodles	3	10.0	4.7%	0.5	27.5	9.0	9.4%	6.0%	1.5%	0%	0.0	0.0%	0.0%	0.0%	30%	6.3	0.0
Sausages	1	17.0	1.9%	0.3	27.5	6.2	6.5%	4.1%	1.0%	0%	0.0	0.0%	0.0%	0.0%	30%	4.3	0.0
Dried fish	1	8.2	3.3%	0.3	27.5	5.1	5.4%	3.4%	0.9%	0%	0.0	0.0%	0.0%	0.0%	30%	3.6	0.0
Cheese (feta type)	1	6.5	3.5%	0.2	27.5	4.4	4.6%	2.9%	0.7%	0%	0.0	0.0%	0.0%	0.0%	30%	3.1	0.0
Cheese (hard)	1	6.5	1.9%	0.1	27.5	2.4	2.5%	1.6%	0.4%	0%	0.0	0.0%	0.0%	0.0%	30%	1.6	0.0
**For all selected products**		**412.8**		**12.0**		**271.4**	**285.7%**	**180.9%**	**45.2%**		**133.3**	**140.3%**	**88.9%**	**22.2%**		**190.0**	**93.3**
**For all products except HH salt**		**412.8**		**12.0**		**271.4**	**285.7%**	**180.9%**	**45.2%**		**133.3**	**140.3%**	**88.9%**	**22.2%**		**190.0**	**93.3**
